# CO_2_ Adsorption by Amino-Functionalized Graphene–Silica Gels

**DOI:** 10.3390/gels11090702

**Published:** 2025-09-02

**Authors:** Marina González-Barriuso, Ángel Yedra, Carmen Blanco

**Affiliations:** 1 Materials Chemistry Group, Department QuIPRe, Universidad de Cantabria, Av. de los Castros 46, 39005 Santander, Spain; 2 Fundación Centro Tecnológico de Componentes, C/Isabel Torres 1, 39011 Santander, Spain

**Keywords:** graphene, graphene oxide, mesostructure, silica gels, sol–gel, amine functionalization, surfactant removal, CO_2_ capture

## Abstract

This work evaluates the CO_2_-adsorption relevance and cycling stability of graphene oxide–silica (GO-SiO_2_) and reduced graphene oxide–silica (rGO-SiO_2_) gels after amine functionalization, demonstrating high-capacity retention under repeated adsorption–desorption cycles: rGO-SiO_2_-APTMS retains ≈96.3% of its initial uptake after 50 cycles, while GO-SiO_2_-APTMS retains ≈90.0%. The use of surfactants to control the organization of inorganic and organic molecules has enabled the development of ordered mesostructures, such as mesoporous silica and organic/inorganic nanocomposites. Owing to the outstanding properties of graphene and its derivatives, synthesizing mesostructures intercalated between graphene sheets offers nanocomposites with novel morphologies and enhanced functionalities. In this study, GO-SiO_2_ and rGO-SiO_2_ gels were synthesized and characterized by X-ray diffraction (XRD), differential scanning calorimetry (DSC), thermogravimetric analysis (TG), mass spectrometry (MS), N_2_ adsorption–desorption isotherms, and transmission electron microscopy (TEM). The resulting materials exhibit a laminar architecture, with mesoporous silica domains grown between graphene-based layers; the silica contents are 83.6% and 87.6%, and the specific surface areas reach 446 and 710 m^2^·g^−1^, respectively. The laminar architecture is retained regardless of the surfactant-removal route; however, in GO-SiO_2_ obtained by solvent extraction, a fraction of the surfactant remains partially trapped. Together with their high surface area, hierarchical porosity, and amenability to surface functionalization, these features establish amine-grafted graphene–silica gels, particularly rGO-SiO_2_-APTMS, as promising CO_2_-capture adsorbents.

## 1. Introduction

Graphene and its derivatives, such as graphene oxide (GO), reduced graphene oxide (rGO), and graphene nano-platelets, exhibit properties that make them suitable for numerous applications [1,2]. Table 1 presents a summary of these applications with examples. Combining these properties with ordered mesostructures gives rise to a new kind of composite [3,4,5,6,7,8,9]. Ordered mesostructures are commonly synthesized using surfactants as templates to direct structural organization.

Composites that couple graphene derivatives with ordered mesostructures have been explored for different applications depending on their composition and final structure. For example, titanium dioxide (TiO_2_)–graphene aerogel composites are synthesized as anode materials for lithium-ion batteries [6]. TiO_2_ is an abundant Li-ion insertion material that undergoes less than ~4% volume expansion during cycling, making it attractive as an anode. However, TiO_2_ polymorphs possess relatively low theoretical capacity, electronic conductivity, and Li-ion diffusion coefficients. Incorporation of graphene into TiO_2_ mesostructures mitigates these drawbacks. Sulfonate graphene–N-doped mesoporous carbons composites have been used for supercapacitor applications [7]. Graphene is a promising supercapacitor–electrode material owing to its high electrical conductivity, large specific surface area, and chemical stability, while ordered mesoporous silica provide well-controlled porosity and a large surface area; their combination prevents graphene sheet agglomeration and leverages the advantages of both materials. Furthermore, mesoporous graphene oxide/SBA-15 nanocomposites have been developed for water purification [8]. GO is an effective adsorbent for removing organic and inorganic species from water; however, its hydrophilicity and the difficulty in filtration and reuse limit practical application, necessitating a support such as SBA-15. Another application of graphene structures is CO_2_ adsorption: amine-modified silica–reduced graphene oxide composites have been investigated for this purpose [9], where graphene derivatives are incorporated into a silica framework to improve thermal stability.

Herein, graphene oxide–silica (GO-SiO_2_) and reduced graphene oxide–silica (rGO-SiO_2_) gels are prepared by the sol–gel method. Once functionalized with amine groups, these materials are intended for CO_2_ capture. For such processes, both silica-based and carbonaceous materials have been studied [20,21,22,23]. Combining them into graphene–silica gels is expected to improve CO_2_ capture performance. In this study, silica was intercalated between the laminar structure of GO. The mesoporous silica was confined between the adjacent flat surfaces of the GO, reinforcing its mesostructure. For instance, these mesostructures are used for environmental remediation applications. This confinement between the sheets allowed the mesoporous silica to intercalate compactly between two sheets of these carbon structures, developing mesoporous material in the galleries of both graphene derivatives. The synthesized samples were characterized, and their results were compared.

Removal of the surfactant used to template these materials is a critical step because improper removal compromises textural parameters. This is particularly important if the chemical characteristics of the GO (notably, the hydroxide and acid groups) must be preserved. For GO-SiO_2_, the surfactant was removed by solvent extraction [24,25], whereas for rGO-SiO_2_ it was eliminated by calcination [26,27,28]. These calcination processes achieved a reduction in GO and surfactant elimination in a single step.

## 2. Results and Discussion

### 2.1. Characterization of rGO-SiO_2_ and GO-SiO_2_ Gels

**Figure 1:**
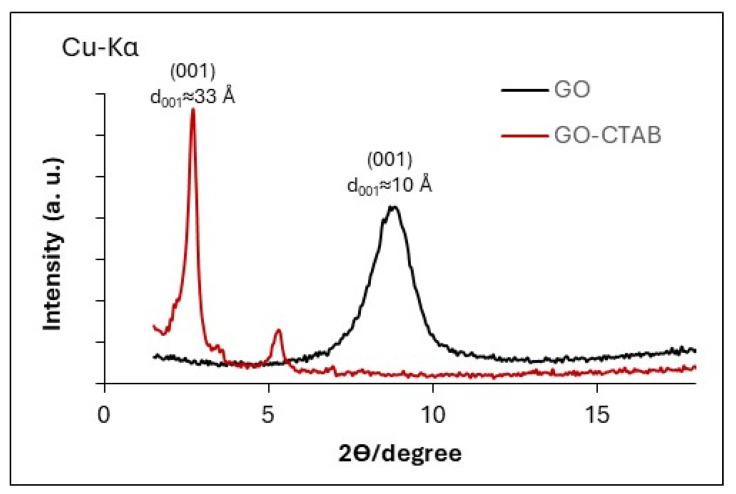
XRD patterns of GO (graphene oxide) and GO–CTAB (graphene oxide treated with CTAB). The basal reflection is indexed as (001); d_001_ increases from ~10 Å (GO, 2θ ≈ 8.8°, Cu Kα) to ~33 Å (GO–CTAB, 2θ ≈ 2.7°) upon CTAB intercalation, evidencing gallery expansion.

XRD was used to examine whether the basal spacing between the GO sheets changes upon intercalation of the template (CTAB). Figure 1 shows the XRD patterns for GO and GO-CTAB. The presence of diffraction peaks confirms the laminar structure of both samples. After the CTAB intercalation, the basal spacing increases. According to Bragg’s law, the GO-CTAB sample exhibits a basal spacing of 33 Å prior to subsequent hydrolysis with either silica source, whereas pristine GO shows 10 Å.

**Figure 2:**
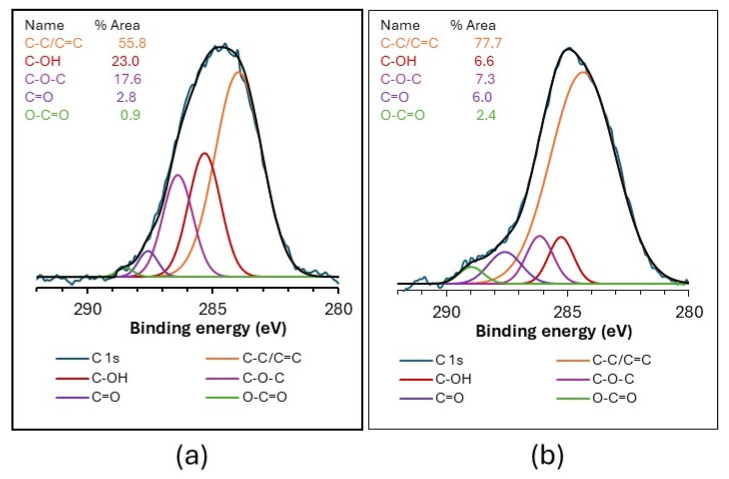
High-resolution C 1s XPS spectra of GO-SiO_2_ (graphene oxide–silica gel) (**a**) and rGO-SiO_2_ (reduced graphene oxide–silica gel) (**b**).

Figure 2 shows the deconvolution of the high-resolution C 1s XPS spectra of the GO-SiO_2_ and rGO-SiO_2_ samples. The chemical state and functional groups can be evaluated from these spectra. The deconvoluted C 1s components are assigned to C-C/C=C bonds (284.0 and 284.4 eV), C-OH (285.3 eV), C-O-C (286.4 and 286.2 eV), C=O (287.6 eV), and O-C=O (288.5/289.0 eV) [29,30,31]. Each component contributes a given percentage of the total C 1s area, reflecting the fraction of carbon in that functional environment. As expected, GO-SiO_2_ contains a higher proportion of oxygenated carbon groups than rGO-SiO_2_ (44.2% vs. 22.3%), consistent with the partial reduction in GO during the thermal treatment used to prepare rGO-SiO_2_. This thermal treatment was not applied to GO-SiO_2_ because CTAB was removed by solvent extraction.

**Figure 3:**
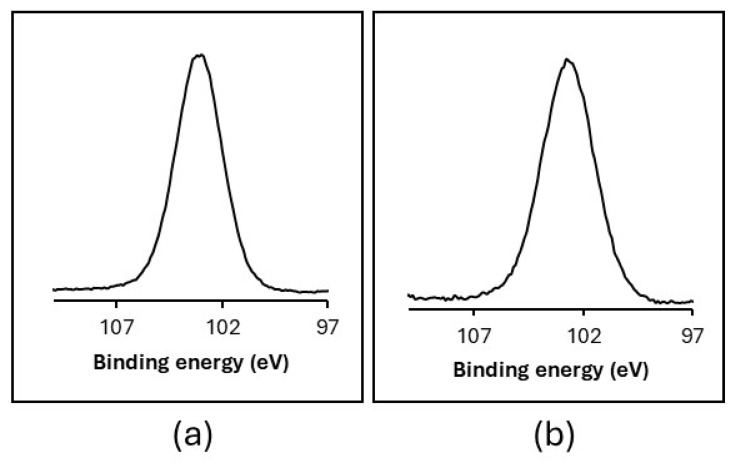
High-resolution Si 2p XPS spectra of GO-SiO_2_ (**a**) and rGO-SiO_2_ (**b**).

The high-resolution Si 2p XPS spectra of GO-SiO_2_ and rGO-SiO_2_ show a single component at 103.0 eV attributable to a Si-O bond [31] (Figure 3). Together with the C 1s spectra data, this indicates no detectable covalent interactions between carbons in the graphene structures and silicon in the silica.

The Si 2p region shows no measurable shift between GO-SiO_2_ and rGO-SiO_2_, with a single Si–O component at 103.0 eV in both samples, indicating an unchanged silica environment and no evidence of Si–C bond formation. In the C 1s region, rGO-SiO_2_ exhibits a slight positive shift of the graphitic C–C/C=C component (284.4 eV vs. 284.0 eV in GO-SiO_2_), a small negative shift of C–O–C (286.2 eV vs. 286.4 eV), and a modest positive shift of O–C=O (289.0 eV vs. 288.5 eV). These changes (ΔBE ≈ +0.4, −0.2, and +0.5 eV, respectively) are consistent with a partial reduction in GO upon calcination (altering the local electronic environment and redistributing oxygenated functionalities), together with minor differential charging expected for these insulating, silica-rich gels. Overall, the spectra reflect a reorganization of surface oxygen groups rather than the emergence of new silicon–carbon bonding.

**Figure 4:**
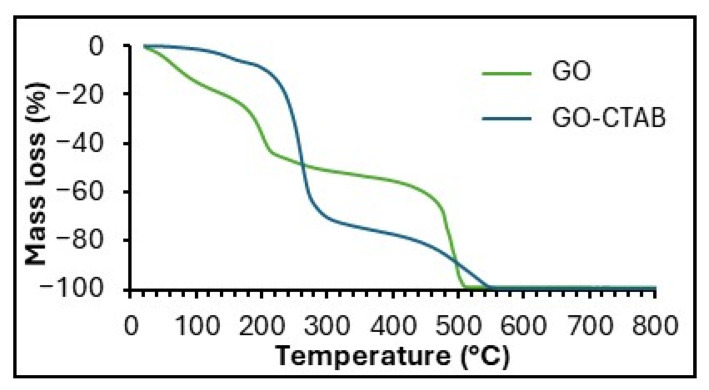
TG curves of GO and GO-CTAB.

Figure 4 presents the TG curves of GO and GO-CTAB, which exhibit an initial mass loss of up to 100 °C due to desorption of water, a second mass loss around 220 °C associated with degradation of oxygen-containing surface groups, and a third mass loss near 510 °C due to combustion of the carbon framework. GO-CTAB also shows a mass loss between 200 and 320 °C corresponding to decomposition of the cetyltrimethylammonium bromide surfactant. In both samples, mass loss progresses to completion by 800 °C.

**Figure 5:**
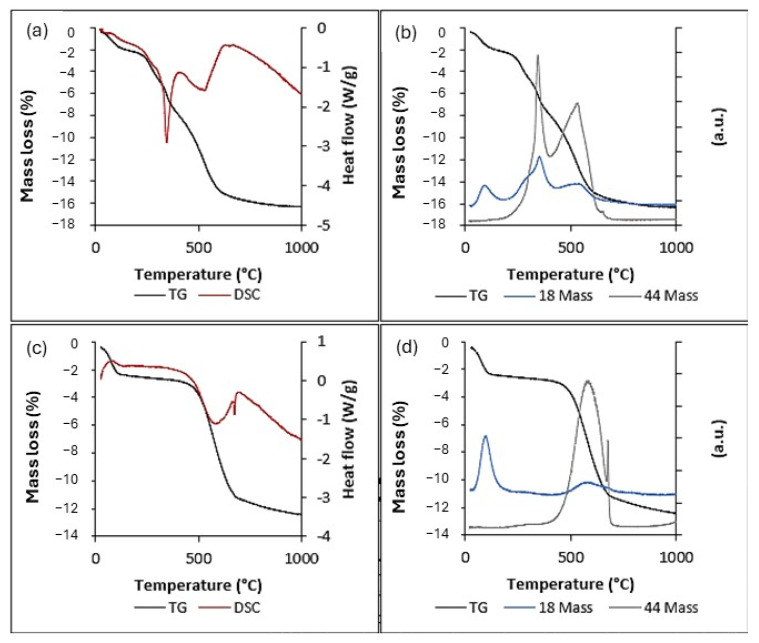
GO-SiO_2_ sample: (**a**) TG (black) and DSC (red); (**b**) TG (black), with MS monitoring at m/z 18 (blue) and 44 (green). rGO-SiO_2_ sample: (**c**) TG (black) and DSC (red); (**d**) TG (black) with monitoring at m/z 18 (blue) and 44 (green).

A thermogravimetric analysis of GO-SiO_2_ is shown in Figure 5. Five mass loss steps are observed. The first, at around 80 °C, corresponds to the loss of adsorbed water (2.0%) and an endothermic peak. Both features are corroborated by the DSC curve and by monitoring m/z 18 (water) and 44 (carbon dioxide) in the MS; see Figure 5a,b, respectively. Three subsequent steps correspond to exothermic peaks with minima at around 280, 340, and 530 °C. These are assigned to (i) decomposition of oxygenated groups on GO at 280 °C (2.4%), which appears as a shoulder in the m/z 18 signal (Figure 5b); (ii) decomposition of residual CTAB at 340 °C (3.6%), consistent with concurrent m/z 18 and 44 signals (Figure 5b); and (iii) decomposition of GO at 530 °C (8.4%), again accompanied by H_2_O and CO_2_ signals (Figure 5b). Finally, an exothermic peak at 640 °C with a CO_2_ MS signal is observed, also attributable to carbon decomposition. This peak is more intense in the rGO-SiO_2_, likely due to carbonaceous residues after thermal surfactant removal. The solid residue from the GO-SiO_2_ analysis is a white powder (83.6%), attributable to the silica content.

Similarly, the rGO-SiO_2_ thermogravimetric analysis (Figure 5c,d) shows two mass loss steps. The first, at around 80 °C, corresponds to desorption of water (2.4%) and an endothermic peak, as corroborated by DSC and MS (m/z 18 and 44). The second (10.0%) encompasses two exothermic peaks with minima near 580 °C and a sharper feature at 700 °C, due to the decomposition of the carbonaceous traces associated with rGO. In this sample, 87.6% remains as an inorganic residue, corresponding to silica.

Comparing the GO-SiO_2_ and rGO-SiO_2_ TG curves indicates that calcination to 380 °C removes CTAB completely from GO-CTAB-SiO_2_, whereas solvent extraction leaves residual surfactant within the pore structure. Moreover, solvent extraction preserves the oxygenated groups of GO, while thermal treatment reduces the surface to rGO.

**Figure 6:**
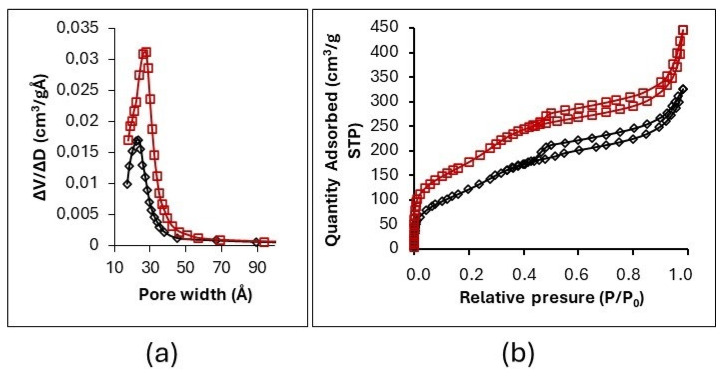
(**a**) BJH pore-size distributions, showing modes at ~23 Å (GO-SiO_2_) and ~27 Å (rGO-SiO_2_). (**b**) N_2_ adsorption–desorption isotherms (type IV(a), H3).

Figure 6b shows the N_2_ adsorption–desorption isotherms and the pore-size distributions of GO-SiO_2_ and rGO-SiO_2_. Both materials exhibit type IV(a) adsorption isotherms, with an H3 hysteresis loop, characteristic of mesoporous adsorbents with slit-like (laminar) pores, as per the IUPAC classification [32,33]. The nearly linear adsorption between relative pressures of 0.03 and 0.25 is consistent with pores in the 15–30 Å range. The pore distribution obtained by the BHJ method (Figure 6a) shows average diameters of 23 Å (GO-SiO_2_) and 27 Å (rGO-SiO_2_), consistent with slit-like mesopores inferred from the H3 hysteresis; the corresponding modal pore diameters (D_pore_) are reported in Table 2.

The GO-SiO_2_ and rGO-SiO_2_ gels display type IV(a) isotherms with H3 hysteresis and mesopores around 20–30 Å (Figure 6), with S_BET_ = 446 and 710 m^2^ g^−1^ and V_pore_ = 0.46 and 0.76 cm^3^ g^−1^ (Table 2), respectively. These metrics are within or above the ranges commonly reported for amine-grafted mesoporous silicas (where grafting usually lowers S_BET_ relative to the parent silica). In contrast, zeolites are predominantly microporous (<1 nm), which can restrict access for bulky organosilanes, while many MOFs offer very high nominal surface areas but largely microporous frameworks and different stability/operability windows.

The specific surface area, average pore size, and N_2_ uptake are larger for rGO-SiO_2_ than for GO-SiO_2_ (Table 2). These differences arise from the amount of surfactant removed by each method, either thermal treatment or solvent extraction. Calcination not only eliminates CTAB but also partially reduces the oxygenated groups on GO, in agreement with the thermogravimetric analysis (Figure 4).

**Figure 7:**
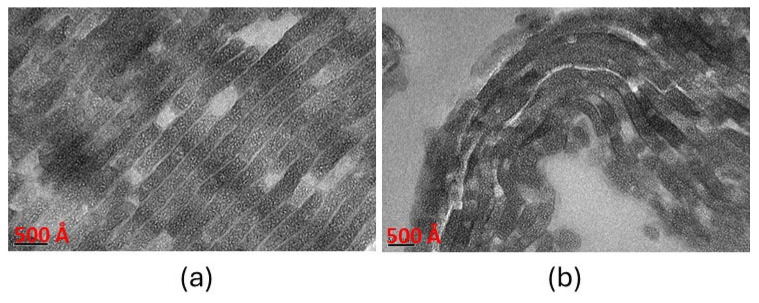
(**a**) TEM image of the GO-SiO_2_ gel; (**b**) TEM image of the rGO-SiO_2_ gel.

TEM images (Figure 7) show that both gels possess a laminar structure with mesopore silica grown between the GO-based sheets. The sheets are arranged parallel to each other, with regular inter-sheet spacing of around 250 Å for GO-SiO_2_ and 230 Å for rGO-SiO_2_. GO-SiO_2_ presents a more regular structure.

### 2.2. Characterization of GO-SiO_2_-APTMS and rGO-SiO_2_-APTMS

**Figure 8:**
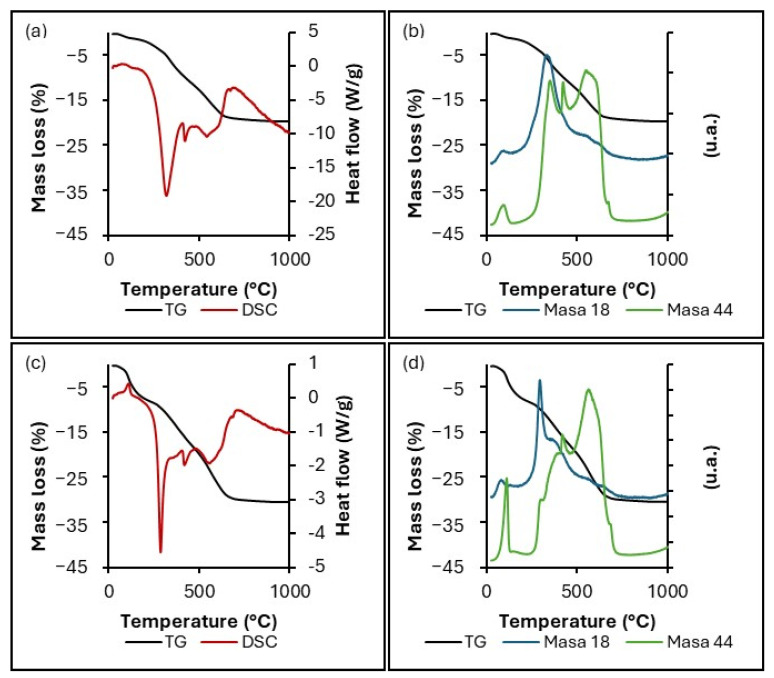
(**a**,**c**) TG (black) and DSC (red) curves; (**b**,**d**) TG (black) with MS monitoring at m/z 18 (blue) and 44 (green) for GO-SiO_2_-APTMS (graphene oxide–silica gel functionalized with APTMS, **top**) and rGO-SiO_2_-APTMS (reduced graphene oxide–silica gel functionalized with APTMS, **bottom**).

Figure 8 compiles the TG-DSC results and MS monitoring at m/z 18 (water) and 44 (CO_2_) for the amino-functionalized samples. Both GO-SiO_2_-APTMS and rGO-SiO_2_-APTMS exhibit an initial mass loss with an endothermic peak at around 90 °C, accompanied by the release of water and CO_2_. Incorporation of APTMS leads not only to desorption of physiosorbed ambient water at this temperature (as observed for the non-functionalized samples, Figure 5) but also to the release of chemisorbed ambient CO_2_ from the surface.

Subsequent mass losses are associated with exothermic peaks, indicating thermal decomposition. For GO-SiO_2_-APTMS, these features arise from the decomposition of oxygen-containing groups on the GO, the decomposition of residual CTAB remaining after ethanol washing, and combustion of the APTMS carbon chain bound to the sample, releasing both water and CO_2_. For the rGO-SiO_2_-APTMS sample, a similar process occurs; however, only the APTMS carbon chain decomposes in this temperature window.

The final mass loss in both amino-functionalized samples, associated with an endothermic minimum around 520–580 °C, corresponds to the thermal decomposition of the reduced graphene oxide framework (in GO-containing samples, GO is thermally reduced during TG). As indicated by MS monitoring at m/z 18 and 44, CO_2_ is predominantly evolved. The white residue corresponds to the silica content, as also observed for the non-functionalized samples.

Table 3 reports the mass loss fraction (wt%) assigned to the combustion of the APTMS alkyl chain covalently grafted to the gels, as extracted from the TG profiles in Figure 8. The table also lists the nitrogen content (N, wt%) and the corresponding amine loading (mmol –NH_2_ g^−1^) for both GO-SiO_2_-APTMS and rGO-SiO_2_-APTMS. The latter values were obtained by combining the percentage of organic matter removed within the specified temperature interval with the stoichiometric relation between the atomic mass of nitrogen and the molar mass of APTMS (one N per APTMS unit), assuming that the organic mass loss in that interval arises predominantly from the grafted organosilane.

**Figure 9:**
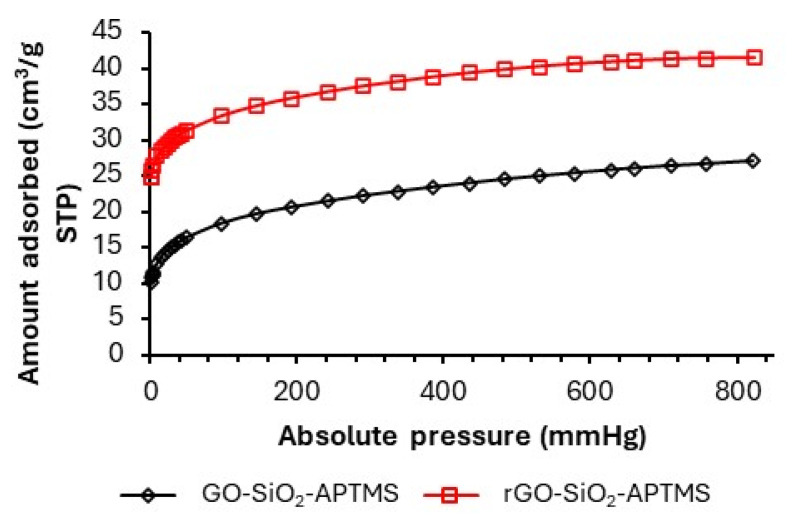
CO_2_ adsorption isotherms at 25 °C of GO-SiO_2_-APTMS and rGO-SiO_2_-APTMS.

Figure 9 shows the CO_2_ adsorption isotherms at 25 °C for GO-SiO_2_-APTMS and rGO-SiO_2_-APTMS. Both display type I behavior, concave with respect to the x-axis (absolute pressure), with uptake tending to level off at higher pressures, typically associated with monolayer adsorption dominated by chemisorption. In this context, CO_2_ reacts with amine groups on the surface to form carbamate species according to the following equilibrium:

RNH_2_ + CO_2_ ⇆ RNHCO_2_H

This chemisorption mechanism is desirable for carbon capture applications because it implies strong and selective binding of CO_2_ at low partial pressures, as encountered in post-combustion gas streams. Type I isotherms also suggest an energetically homogeneous set of adsorption sites, beneficial for predictable performance in practical adsorption systems.

**Figure 10:**
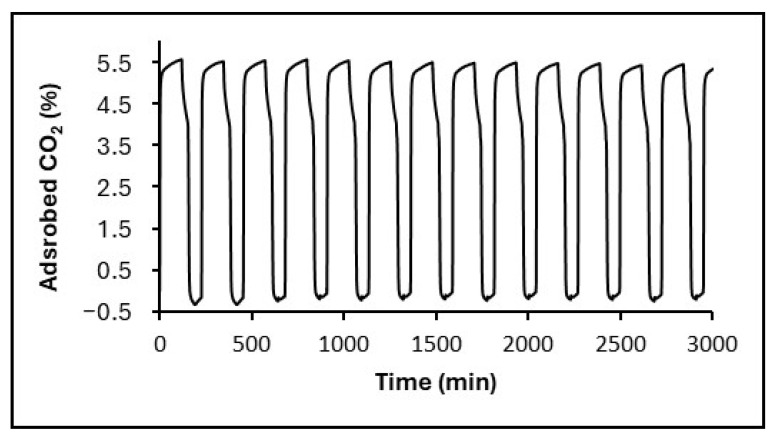
Representative isothermal CO_2_ capture–regeneration cycles at 25 °C for rGO-SiO_2_-APTMS under a 20% CO_2_/N_2_ stream (adsorption) and N_2_ at 110 °C (regeneration). A rapid initial uptake is followed by a slower approach to equilibrium within each cycle; capacity retention over 50 cycles is reported in Table 6.

At 25 °C, Table 4 benchmarks the CO_2_ uptake at 1.0 atm and 0.1 atm for the two graphene–silica gels alongside a silica-only reference gel (SiO_2_-APTMS), synthesized analogously to the present materials but without the graphene component. rGO-SiO_2_-APTMS exhibits the highest absolute uptake (41.4 ± 0.4 and 32.3 ± 0.3 cm^3^ g^−1^ at 1.0 and 0.1 atm, respectively), followed by SiO_2_-APTMS (39.8 ± 0.4 and 29.3 ± 0.3 cm^3^ g^−1^), whereas GO-SiO_2_-APTMS is lower (26.6 ± 0.3 and 17.3 ± 0.2 cm^3^ g^−1^). The near-parity between rGO-SiO_2_-APTMS and the silica-only reference, despite the former’s lower amine loading (Table 3), indicates improved site accessibility within the laminar rGO framework, consistent with the complete removal of CTAB during calcination and the concomitant reduction in steric hindrance across the mesoporous network. When contextualized against state-of-the-art adsorbents, the amine-grafted rGO-SiO_2_ gels combine mesoporous accessibility with robust cycling, yielding CO_2_ uptakes at 25 °C that are on par with typical amine-functionalized mesoporous silicas under low-pressure conditions while delivering higher per-site utilization (Table 5) and excellent retention after 50 cycles (Table 4, Figure 10). In contrast to predominantly microporous frameworks such as zeolites and many MOFs, the laminar mesopores (≈20–30 Å) in our gels facilitate amine accessibility and diffusion, which is directly reflected in the per-site efficiencies reported here.

Accordingly, GO-SiO_2_-APTMS shows lower CO_2_ uptake (26.6 ± 0.3 and 17.3 ± 0.2 cm^3^ g^−1^ at 1.0 and 0.1 atm, respectively) and a lower per-site efficiency (59.4%) than rGO-SiO_2_-APTMS (41.4 ± 0.4; 32.3 ± 0.3 cm^3^ g^−1^; 81.0%), and it retains ≈90.0% of its initial capacity after 50 cycles versus ≈ 96.3% for rGO-SiO_2_-APTMS under the same regeneration protocol.

Using the amine loading reported in Table 3 (mmol –NH_2_ g^−1^), together with the CO_2_ uptake reported in Table 4 (converted to a molar basis on the same mass of adsorbent), the per-site chemisorption efficiency can be calculated as

η = n(CO_2_)/n(−NH_2_) × 100%

assuming a 1:1 (mol–mol) stoichiometry between CO_2_ and the accessible amine groups under the present conditions. As summarized in Table 5, the calculated efficiencies indicate that rGO-SiO_2_-APTMS exhibits a higher CO_2_-capture efficiency per amine site than GO-SiO_2_-APTMS and SiO_2_-APTMS, consistent with the improved textural accessibility inferred from the nitrogen sorption data.

In GO–SiO_2_, the exothermic mass loss at ~340 °C with concurrent m/z 18 and 44 signals (Figure 5a,b) is assigned to residual CTAB after solvent extraction; consistent with the N_2_-sorption data (Figure 6, Table 2), this residual surfactant partially occludes mesopores and limits subsequent amine site accessibility. Taken together, these textural effects rationalize the lower uptake and per-site efficiency of GO-SiO_2_-APTMS relative to rGO-SiO_2_-APTMS (Table 4 and Table 5) and the slightly lower capacity retention during cycling (Figure 10).

### 2.3. Cyclic Adsorption and Desorption of CO_2_

To evaluate stability toward CO_2_ capture under repeated operation, we performed cyclic adsorption–desorption tests combining thermogravimetric cycling and extended fixed-bed runs (see Section 4.4). Table 6 compiles the results of the CO_2_ adsorption–desorption cycling. In addition to the composites, an amino-functionalized mesoporous silica sample (SiO_2_-APTMS), synthesized analogously but without the graphene-based materials, is included. The first notable observation is that rGO-SiO_2_-APTMS adsorbs more CO_2_ than GO-SiO_2_-APTMS, with an increase of 2.4%, consistent with the CO_2_ adsorption–desorption isotherms discussed in Figure 9. SiO_2_-APTMS shows a CO_2_ adsorption capacity similar to that of the samples under study.

For rGO-SiO_2_-APTMS and GO-SiO_2_-APTMS, the mass percentage of adsorbed CO_2_ remains nearly constant up to cycle 15, with a slight decrease in cycles 25 and 50. This translates into retentions of 96.3% and 90,0%7 for the initial capacity at cycle 50, respectively. In contrast, for GO-SiO_2_-APTMS, the adsorbed amount remains stable only during the first three cycles and then progressively decreases. Compared with the sample lacking graphene-based material (SiO_2_-APTMS), the adsorption capacity drops even further, down to 76.6%. Therefore, introducing the graphene-based material enhances stability over CO_2_ adsorption–desorption cycles, with a more pronounced improvement when the graphene material is in its reduced form.

As an example of the behavior during the first fourteen isothermal CO_2_ capture–regeneration cycles at 25 °C, Figure 10 shows the corresponding data for rGO-SiO_2_-APTMS. A strong initial CO_2_ uptake is observed in the first minutes of each cycle, attributed to chemisorption via carbamate formation between amine groups and the CO_2_, followed by a continuous increase over 50 min, approaching equilibrium.

As summarized in Table 6 and illustrated in Figure 10, both amine-functionalized gels show minimal capacity fade. rGO-SiO_2_-APTMS retains ≈96.3% of its initial uptake after 50 cycles, whereas GO-SiO_2_-APTMS retains ≈90.0%; by contrast, SiO_2_-APTMS (without the graphene component) retains ≈76.6%. The superior retention of rGO-SiO_2_-APTMS is consistent with its higher accessible surface area and reduced residual surfactant, improving amine site accessibility throughout the laminar mesoporous framework.

### 2.4. Kinetics and Thermodynamics of CO_2_ Capture Reaction

As outlined in Section 2.2, CO_2_ uptake proceeds via the CO_2_–amine surface reaction, leading to ammonium–carbamate (dry). On this basis, below, we discuss the apparent kinetic order in CO_2_ and benchmark its rate constants, as well as the literature values of ΔH and ΔS, to place our 25 °C isotherms of GO-SiO_2_-APTMS and rGO-SiO_2_-APTMS in context.

In solid-supported primary amines, CO_2_ adsorption proceeds predominantly via ammonium–carbamate ion pairs under dry conditions, while bicarbonate–carbonate species become significant in the presence of water [34,35]. This mechanistic picture (zwitterion formation followed by deprotonation by a neighboring amine) is consistently reported across supported-amine systems.

Under the nominally dry conditions used here, CO_2_ capture on primary amines proceeds via the classical zwitterion–deprotonation sequence to form an ammonium–carbamate ion pair, whereas in the presence of water, a bicarbonate route can also contribute, effectively requiring only one amine per CO_2_. Consistent with this framework, the higher per-site efficiency observed for rGO-SiO_2_-APTMS (Table 5) reflects improved site accessibility within the laminar rGO scaffold and general–base assistance by neighboring amines and/or silanols that facilitates the deprotonation step.

At 25 °C, and at low surface coverage, the apparent reaction order in CO_2_ is typically close to the first order (m ≈ 0.8–1.0), decreasing toward zero as the surface approaches saturation. Transient uptakes on amine-grafted silica are commonly fitted with pseudo-first-order or Avrami models, which capture the exponential-to-fractal approach to equilibrium; reported apparent rate constants at 25 °C span 10^−4^–10^−2^ s^−1^, depending on amine type/loading, porosity, gas residence time, and humidity [36]. These trends are consistent with an Eley–Rideal-type event [37] between gaseous CO_2_ and available surface –NH_2_ sites.

Regarding thermodynamics, multi-temperature studies on amine-functionalized silica generally indicate exothermic adsorption, with ΔH_ads_ for primary amines in dry conditions in the −70 to −100 kJ mol^−1^ range (chemisorption), while humid conditions and/or higher coverages often yield apparent heats in the −30 to −60 kJ mol^−1^ range due to changes in speciation and competitive adsorption [38]. The entropy change is negative (≈−50 to −150 J mol^−1^ K^−1^), reflecting ordering upon ion pair formation [38]. For GO/rGO-SiO_2_ hybrids, the literature indicates that the graphene-derived microenvironment can modulate both kinetics and apparent heats, often improving uptake or mass transfer characteristics relative to bare silica [39].

Because the present work reports single-temperature isotherms at 25 °C, we refrain from extracting system-specific ΔH/ΔS values, which require multi-temperature datasets (Van ’t Hoff) or microcalorimetry. Instead, we benchmark our materials within the above literature ranges, which are mechanistically consistent with the shape of our 25 °C isotherms and with the chemistry of APTMS-grafted silica and GO/rGO-SiO_2_ composites.

## 3. Conclusions

Two types of laminar mesoporous gels were formed: GO-SiO_2_ and rGO-SiO_2_. Both present regular interplanar spacing between GO-based sheets, with mesopore silica filling these spaces; the silica contents are 83.6 and 87.6%, respectively. The nature of the sheets differs because of the surfactant removal method used during synthesis: solvent extraction preserves the GO character, whereas thermal elimination reduces GO to rGO, changing its chemical nature. This change involves the partial elimination of the oxygenated groups on its surface. Calcination eliminates the surfactant completely, unlike solvent extraction, which leaves residual surfactant within the structure. This is reflected in N_2_ adsorption–desorption measurements, where textural parameters are higher for rGO-SiO_2_.

Amine functionalization of these materials was confirmed by combined TG–DSC analysis and CO_2_ adsorption isotherm. The amine-functionalized gels with the highest CO_2_ adsorption capacity are those containing reduced graphene oxide (rGO). Moreover, regeneration cycle data indicate that these composites exhibit greater stability of adsorption capacity upon repeated cycling. The CO_2_ adsorption isotherms indicate that adsorption occurs predominantly via chemisorption, attributed to the reaction between CO_2_ and primary amine sites, which forms carbamates. 

Importantly, cyclic tests confirm excellent stability toward CO_2_ capture: rGO-SiO_2_-APTMS retains ≈96.3% of its initial uptake after 50 cycles, outperforming GO-SiO_2_-APTMS (≈90.0%) and SiO_2_-APTMS (≈76.6%).

Complete surfactant removal in the rGO-derived gels enhances textural accessibility and translates into higher CO_2_ uptake, greater per-site efficiency, and slightly better capacity retention than in the GO-derived gels, where residual CTAB partially occludes mesopores.

In sum, rGO-SiO_2_-APTMS couples competitive mesoporous textural metrics for an amine-grafted silica with high per-site efficiency and robust cycling, underscoring its competitiveness relative to conventional silica adsorbents and its practical advantages versus predominantly microporous frameworks.

## 4. Materials and Methods

### 4.1. Materials

GO was supplied by Graphenea (San Sebastián, Spain). Cetyltrimethylammonium bromide (CTAB), sodium hydroxide (NaOH, 99%), dodecylamine (98%), tetramethylammonium hydroxide (TMAOH, 98%), tetraethyl orthosilicate (TEOS), fumed silica (98%), and a sodium silicate solution (SiO_2_ 26.5–Na_2_O 10.6%) were purchased from Sigma Aldrich (St. Louis, MO, USA).

### 4.2. Synthesis

First, CTAB was intercalated between GO sheets, following the protocol of Wei [3]. Briefly, 2 g of CTAB was dissolved in 40 mL of 0.1 N NaOH. Then, 0.2 g of GO was added, and the mixture was ultrasonicated, followed by stirring for 5 days at room temperature. The resultant suspension was filtered and washed with deionized water. The solid (GO-CTAB) was dried at 60 °C for 3 days.

In a second step, the dried GO-CTAB was stirred in a 1.03% (*w*/*w*) aqueous dodecylamine solution for 3 h at 50 °C. Then, 8 mL of TEOS was added, and the suspension was stirred for 5 h at 50 °C. The resulting material (GO-CTAB-SiO_2_) was centrifuged and dried at 60 °C for 7 days.

**Figure 11:**
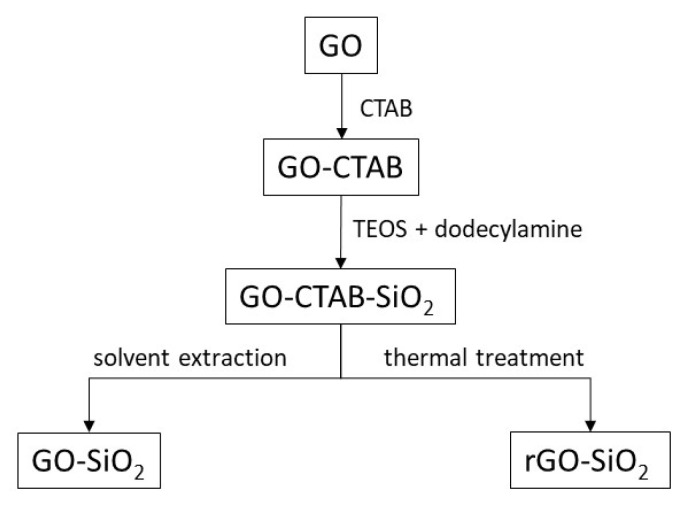
Synthesis diagram of GO-SiO_2_ and rGO-SiO_2_ gels.

Finally, the surfactant template (CTAB) was removed; two different methods were used: thermal treatment and solvent extraction. The thermal treatment consisted of heating GO-CTAB-SiO_2_ in air at 10 °C/min up to 380 °C, followed by a 30 min isotherm; the product was labeled rGO-SiO_2_. Solvent extraction was carried out with ethanol (80 mL of ethanol per gram of GO-CTAB-SiO_2_) at reflux for 3 days; the solid was filtered, washed, and dried at 65 °C. This extraction was performed twice; the sample was labeled GO-SiO_2_. Figure 11 shows a diagram summarizing these syntheses.

GO-SiO_2_ and rGO-SiO_2_ gels were functionalized with (3-aminopropyl)trimethoxysilane (APTMS).

In a typical procedure, 0.5 g of the composite was dispersed in 25.0 mL of anhydrous toluene under magnetic stirring. APTMS was added at 5 mmol per gram of gel. The suspension was refluxed under a nitrogen atmosphere for 24 h. The solid product was recovered by vacuum filtration, washed thoroughly with toluene, and dried at 60 °C for 24 h.

The resulting materials were labeled as GO-SiO_2_-APTMS and rGO-SiO_2_-APTMS.

A mesoporous silica reference functionalized with APTMS (SiO_2_-APTMS) was synthesized analogously to the target gels, following the rGO-SiO_2_-APTMS protocol but omitting the graphene component.

### 4.3. Characterization

XRD was performed on a Bruker D8 Advance diffractometer (Billerica, MA, USA) using Cu Kα radiation.

XPS was carried out using a SPECS Phoibos 100 MCD5 hemispherical electron analyzer (Berlin, Germany) operated in constant energy mode with a Kα Mg X-ray source and an electron flood gun to compensate for sample charging effects. Samples were dried under vacuum prior to analysis.

DSC and TG were performed on a Setaram TG-DSC instrument (Caluire-et-Cuire, France) from room temperature to 1000 °C in air, at a heating rate of 10 °C/min and an air flow of 50 cm^3^·min^−1^.

MS was carried out with a ThermoStar GSD 301 T instrument from Pfeiffer Vacuum (Aβlar, Germany) connected in series to the TG-DSC Setaram. The operating pressure ranged from 1·10^−6^ to 5·10^−6^ mbar.

N_2_ adsorption–desorption isotherms were recorded on a Micromeritics ASAP-2000 volumetric gas adsorption analyzer at the saturation temperature of nitrogen, over relative pressures of 0 to 1. Prior to measurement, samples were outgassed at 140 °C and 10^−4^ mbar for 16 h. Specific surface areas were calculated using the Brunauer–Emmett–Teller (BET) method. Total pore volume (V_pore_) was taken as the volume of liquid N_2_ adsorbed at a relative pressure close to unity (P/P^0^ = 0.95), calculated according to Gurvitch’s rule. Pore size distributions were obtained using the Barrett–Joyner–Halenda (BJH) method for mesoporous materials [40].

TEM was performed on a JEOL JEM-2100 microscope (Peabody, MA, USA). Samples were prepared using conventional procedures and sectioned into electron-transparent slices by ultramicrotomy.

### 4.4. CO_2_ Capture Evaluation

CO_2_ adsorption isotherms were obtained on a Micromeritics ASAP-2000 volumetric analyzer at 25 °C, over relative pressures of 0 to 1. Before measurement, samples were outgassed at 140 °C and 10^−4^ mbar for 16 h.

TG cycling was used to analyze cyclic CO_2_ (adsorbate) adsorption and desorption by the adsorbents developed in this study, using a SETARAM thermobalance. Gas flow rate, temperature, and cycle duration were optimized beforehand. Residual moisture and CO_2_ were first removed by purging with nitrogen gas (N_2_) at 50 cm^3^·min^−1^ while heating from room temperature to 110 °C at 10 °C·min^−1^. Subsequently, a N_2_/CO_2_ (4:1, 20% CO_2_) stream was introduced into the thermobalance, and the sample mass was monitored for a 5 h period to evaluate CO_2_ capture capacity. Desorption was then induced by switching back to pure N_2_ and heating to 110 °C at a rate of 10 °C·min^−1^, holding for 1 h, and cooling back to room temperature.

**Figure 12:**
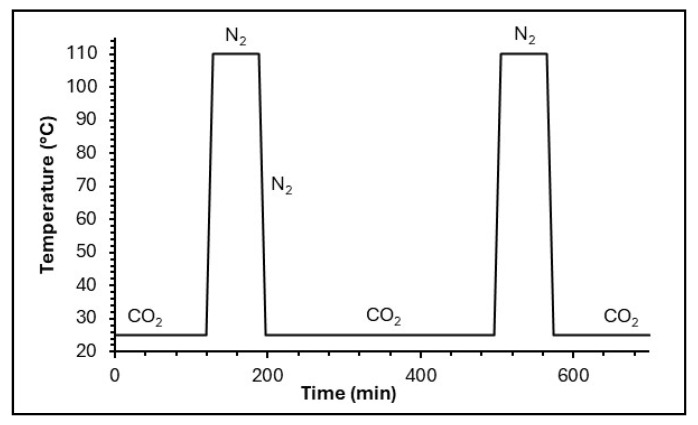
Diagram of the TG cycle process.

These CO_2_ adsorption–desorption cycles were repeated up to six times to assess the stability (Figure 12).

For extended cycling (15, 25, or 50 cycles), the samples were first cycled in a fixed-bed microreactor, following the same protocol (exposure to a N_2_/CO_2_ gas mixture (20% CO_2_) for 5 h, followed by regeneration at 110 °C in N_2_, heating, 1 h isotherm, and cooling), repeated 15, 25, and 50 times.

The samples were then quantitatively analyzed in the thermobalance: an initial N_2_ cycle to clean the surface, followed by a full N_2_/CO_2_ (20%) cycle to determine the CO_2_ capture capacity after the fixed-bed cycling.

## Figures and Tables

**Table 1:** Representative applications of graphene and its derivatives.

Application	Material	Reference
Batteries	Titanium dioxide (TiO_2_)–graphene aerogel composites	[6]
	Laser-induced graphene	[10]
	Graphene nanosheets–Li_2_S composites	[11]
	Fluorinated graphene oxide	[12]
	ZrN@reduced graphene oxide composite	[13]
Supercapacitors	Sulfonate graphene–N-doped mesoporous carbon composites	[7]
	GO/ZnO	[14]
	Fe_2_O_3_/rGO	[15]
	MXene–graphene oxide composite	[16]
Water purification	Mesoporous graphene oxide–SBA-15 nanocomposites	[8]
	GO-CNT–AgI nanocomposite	[17]
	Anthraquinone-2-carboxylic acid on aminated graphene sheets	[18]
	Graphene oxide membrane	[19]
CO_2_ adsorption	Amine-modified silica–reduced graphene oxide composites	[9]
	Modified graphene	[20]

**Table 2:** Textural parameters (S_BET_, V_pore_, BJH modal pore diameter, D_pore_) and SiO_2_ content of GO-SiO_2_ and rGO-SiO_2_.

Sample	S_BET_ (m^2^ g^−1^)	V_pore_ (cm^3^ g^−1^)	D_pore_ (Å)	SiO_2_%
GO-SiO_2_	446	0.46	23	83.6
rGO-SiO_2_	710	0.76	27	87.6

**Table 3:** Quantification of grafted APTMS in GO-SiO_2_-APTMS and rGO-SiO_2_-APTMS: TG-derived organic mass loss (wt%), nitrogen content (wt%), and amine loading (mmol –NH_2_ g^−1^).

Sample	TG-Derived Organic Mass Loss (wt%)	Nitrogen Content (wt%)	Amine Loading (mmol −NH_2_ g^−1^).
GO-SiO_2_-APTMS	7.54	1.82	1.30
rGO-SiO_2_-APTMS	10.33	2.49	1.78

**Table 4:** CO_2_ adsorption capacity of GO-SiO_2_-APTMS, rGO-SiO_2_-APTMS, and SiO_2_-APTMS (silica gel functionalized with APTMS) at 1 atm and 0.1 atm, derived from CO_2_ adsorption isotherms at 25 °C.

Sample	CO_2_ (1 atm) (cm^3^ g^−1^)	CO_2_ (0.1 atm) (cm^3^ g^−1^)
SiO_2_-APTMS	39.8 ± 0.4	29.3 ± 0.3
GO-SiO_2_-APTMS	26.6 ± 0.3	17.3 ± 0.2
rGO-SiO_2_-APTMS	41.4 ± 0.4	32.3 ± 0.3

**Table 5:** Efficiency of the chemisorption reaction for carbamate formation.

Sample	Amine Loading (mmol –NH_2_ g^−1^)	CO_2_ (0.1 atm) (mmol g^−1^)	% Efficiency (CO_2_/-NH_2_)
SiO_2_-APTMS	2.64	1.3	49.2
GO-SiO_2_-APTMS	1.30	0.77	59.4
rGO-SiO_2_-APTMS	1.78	1.44	81.0

**Table 6:** CO_2_ captured (mass %, per cycle) during adsorption–desorption cycling at 25 °C in 20% CO_2_/N_2_, followed by regeneration in N_2_ at 110 °C. R_50_ denotes capacity retention after 50 cycles.

Sample	CO_2_ Mass (%) Cycle 1	CO_2_ Mass (%) Cycle 2	CO_2_ Mass (%) Cycle 3	CO_2_ Mass (%) Cycle 4	CO_2_ Mass (%) Cycle 5	CO_2_ Mass (%) Cycle 6	CO_2_ Mass (%) Cycle 15	CO_2_ Mass (%) Cycle 25	CO_2_ Mass (%) Cycle 50
SiO_2_-APTMS	4.7	4.5	4.4	4.3	4.2	4.2	4.0	3.8	3.6
GO-SiO_2_-APTMS	3.0	3.0	3.0	2.9	2.9	2.9	2.8	2.7	2.7
rGO-SiO_2_-APTMS	5.4	5.3	5.4	5.4	5.3	5.4	5.3	5.2	5.2

## Data Availability

The data needed to evaluate the conclusions in this paper are present in the manuscript. Additional raw/processed data related to this study may be requested from the author.

## Abbreviations

The following abbreviations are used in this manuscript:

APTMS	(3-aminopropyl) trimethoxy silane
BET	Brunauer–Emmett–Teller
BJH	Barrett–Joyner–Halenda
CTAB	Cetyltrimethylammonium bromide
Dpt	Department
DSC	Differential scanning calorimetry
GO	Graphene oxide
GO-CTAB	Graphene oxide treated with CTAB
GO-CTAB-SiO_2_	Graphene oxide–silica gel with CTAB
GO-SiO_2_	Graphene oxide–silica gel
GO-SiO_2_-APTMS	Graphene oxide–silica gel functionalized with APTMS
IUPAC	International Union of Pure and Applied Chemistry
MCM-41	Mobil Composition of Matter No. 41
MOF	Metal–organic framework
MS	Mass spectrometry
QuIPRe	Chemistry and Process and Resources Engineering
rGO	Reduced graphene oxide
rGO-SiO_2_	Reduced graphene oxide–silica gel
rGO-SiO_2_-APTMS	Reduced graphene oxide–silica gel functionalized with APTMS
SBA-15	Santa Barbara Amorphous-15
SiO_2_-APTMS	Silica gel functionalized with APTMS
TEM	Transmission electron microscopy
TEOS	Tetraethyl orthosilicate
TG	Thermogravimetry
TMAOH	Tetramethylammonium hydroxide
XPS	X-ray photoelectron spectroscopy
XRD	X-ray diffraction
